# SMAD7 Variant rs4939827 Is Associated with Colorectal Cancer Risk in Croatian Population

**DOI:** 10.1371/journal.pone.0074042

**Published:** 2013-09-16

**Authors:** Iva Kirac, Petar Matošević, Goran Augustin, Iva Šimunović, Vedran Hostić, Sven Župančić, Caroline Hayward, Natasa Antoljak, Igor Rudan, Harry Campbell, Malcolm G. Dunlop, Danko Velimir Vrdoljak, Dujo Kovačević, Lina Zgaga

**Affiliations:** 1 Department of Surgical Oncology, University Hospital for Tumours, Sestre milosrdnice University Hospital Centre, Zagreb, Croatia; 2 Department of Surgery, University Hospital Center, Zagreb, Zagreb, Croatia; 3 German Heart Center, Technical University Munich, Munich, Germany; 4 Department of Anaesthesiology, Sestre milosrdnice University Hospital Centre, Zagreb, Croatia; 5 Department of Surgery, Sestre milosrdnice University Hospital Centre, Zagreb, Croatia; 6 Human Genetics Unit, Institute of Genetics and Molecular Medicine, University of Edinburgh, Edinburgh, United Kingdom; 7 Croatian National Institute of Public Health, Zagreb, Croatia; 8 Department of Medical Statistics, Epidemiology and Medical Informatics, Medical School, University of Zagreb, Zagreb, Croatia; 9 Centre for population health sciences, University of Edinburgh, Edinburgh, United Kingdom; 10 Department of Public Health and Primary Care, Trinity College Dublin, Dublin, Ireland; MOE Key Laboratory of Environment and Health, School of Public Health, Tongji Medical College, Huazhong University of Science and Technology, China

## Abstract

**Background:**

Twenty common genetic variants have been associated with risk of developing colorectal cancer (CRC) in genome wide association studies to date. Since large differences between populations exist, generalisability of findings to any specific population needs to be confirmed.

**Aim:**

The aim of this study was to perform an association study between risk variants: rs10795668, rs16892766, rs3802842 and rs4939827 and CRC risk in Croatian population.

**Methods:**

An association study was performed on 320 colorectal cancer cases and 594 controls recruited in Croatia. We genotyped four variants previously associated with CRC: rs10795668, rs16892766, rs3802842 and rs4939827.

**Results:**

SMAD7 variant rs4939827 (18q21.1) was significantly associated with CRC risk in Croatian population. C allele was associated with a decreased risk, odds ratio (OR): 0.70 (95% CI: 0.57-0.85, P=3.5E-04). Compared to TT homozygotes, risk was reduced by 34% in heterozygotes (OR=0.66, 95% CI: 0.47-0.92) and by 52% in CC homozygotes (OR=0.48, 95% CI: 0.33-0.72).

**Conclusion:**

Our results show association of rs4939827 with colorectal cancer risk in Croatian population. The higher strength of the association in comparison to other studies suggests population-specific environmental or genetic factors may be modifying the association. More studies are needed to further describe role of rs4939827 in CRC. Likely reason for failure of replication for other 3 loci is inadequate study power.

## Introduction

Colorectal cancer (CRC) affects over one million people worldwide each year [1]. It is the third most common cancer and fourth leading cause of cancer mortality [2]. Whilst colorectal cancer mortality has been declining over the last two decades in Europe [3], an increase in both mortality and incidence has been reported for Croatia [4]. When compared to other European countries, Croatia is ranked 4^th^ for mortality in both men (age-standardised rate, ASR: 25.4/100,000) and women (ASR: 13.1/100,000), but according to CRC incidence it is ranked 9^th^ in men (ASR: 44.4/100,000) and 15^th^ in women (ASR: 24.3/100,000) [5]. Colorectal cancer is the 2nd most common cancer and 2nd commonest cause of cancer death in Croatia [6]. Screening programs have been initiated on National level in 2005, their full impact is still expected [7].

It has been estimated that over 30% of the variation in colorectal cancer susceptibility is attributable to genetic factors and majority of this seems to be due to multiple low risk mutations [8]. To date, genome-wide association studies (GWAS) have identified 20 independent single nucleotide polymorphisms (SNP) associated with colorectal cancer risk [9-15].

It is known that the contribution of risk alleles to CRC risk may vary between populations, for example due to allele frequencies or specific linkage disequilibrium (LD) structure, or because of particular genetic and environmental backgrounds may modify the effect of the variants [16,17]. Understanding differential effects in different populations may aid in uncovering disease mechanism, and is important for the translation of results to risk prediction in different populations.

The role of risk loci in colorectal cancer occurrence is largely unexplored in Croatian population. In this paper, we set out to investigate the role of four risk loci: rs4939827, rs10795668, rs3802842 and rs16892766, on colorectal cancer risk in Croatian population.

## Methods

In the period from November 2008 to July 2009, a total of 338 colorectal cancer cases were recruited at departments of surgery in two hospitals in Zagreb, Croatia. Although both are located in Zagreb, these tertiary care hospitals attract patients from across the country. Data on age at diagnosis, sex, tumour histology and TNM stage were collected from CRC patients’ medical records and histopathology reports. Eighteen cases were excluded from the analysis because diagnosis of adenocarcinoma was not confirmed in histopathology report. Metastasis identified intraoperatively or confirmed by imaging methods were considered in evaluating disease progression. Colorectal cancer cases were classified into stages according to American Joint Committee on Cancer (AJCC) 7th edition [18].

594 controls were selected from the ‘10,001 Dalmatians’ study, an ongoing genetic study in Croatia. This study includes approximately three thousand individuals from the general population of Split and from Vis and Korčula Islands in Croatia. Controls were picked randomly, but with constrains regarding age (+/- 5 years) and sex. Participants with personal history of malignant disease were excluded from the control group.

Informed consent was obtained in writing from all participants. The study was approved by the appropriate ethics committees: the Ethics Committee of the University of Split Medical School and Ethical Board of the University Hospital Center Sestre milosrdnice.

At recruitment, peripheral blood sample was collected from each subject and stored in EDTA coated vials. DNA was extracted using standard procedures. In cases, variants rs4939827, rs3802842, rs10795668 and rs16892766 were genotyped using the TaqMan SNP Genotyping Assay. DNA samples of controls were genotyped according to the manufacturer’s instructions on Illumina Infinium SNP bead microarrays (HumanHap300v1 or HumanCNV370-Quad). Genotypes were determined using Illumina BeadStudio software. Samples with a call rate below 97% were excluded. Variants rs4939827 and rs3802842 and were represented on the array and directly genotyped while rs10795668 and rs16892766 were imputed. Imputation with reference to HapMap CEU build 36 release 22 was performed using the software MACH v1.0.15 after exclusion of SNP with MAF <0.01, call rate <98% and HWE deviation p <10-6. Imputation quality was 99% for rs10795668 and 98% for rs16892766.

### Statistical Analysis

R software was used for analyses and R package GenABEL was used for manipulating genetic data. χ^2^ test was used to assess deviations from Hardy-Weinberg equilibrium. Age- and sex-adjusted logistic regression analysis of CRC on allele dosage (0, 1 or 2) was performed to assess the association between four variants and colorectal cancer risk, under the assumption of additive genetic model. Multiple testing corrected *P*-value of *P*<0.0125 was considered representative of statistical significance. 

## Results

A total of 320 (40% female) colorectal cancer cases and 594 (45% female) controls were included in this study. Mean age of cases was 68.91 ± 10.08 y and of controls 66.12 ± 10.23 y. Where tumour site was known, 56% of cases had colon cancer and 44% had rectal or rectosygmoid tumour. Majority of cases had disease in AJCC stages 2 and 3 (33% and 42%, respectively). The characteristics of cases and controls are detailed in Table **1**.

**Table 1:** Characteristics of study population.

**Variable**		**Cases**	**Controls**
Number		320 (35%)	594 (65%)
Sex			
	Male	192 (60%)	324 (55%)
	Female	128 (40%)	270 (45%)
Age (years)		68.91 ± 10.08	66.12 ± 10.23
AJCC stage			
	I	56 (18%)	
	II	107 (33%)	
	III	134 (42%)	
	IV	21 (7%)	
	Unknown	2 (1%)	
	Total	320 (100%)	
Site			
	Colon	128 (40%)	
	rectosigmoid	7 (2%)	
	Rectum	94 (29%)	
	Unknown	91 (28%)	
	Total	320 (100%)	
Tumour size (cm)		5.0 ± 2.1	
	range (cm)	1-13	
Lymph nodes ^a^			
	Sampled	15 (IQR: 14-19)	
	positive (%)	5 (IQR: 0-27)	
	positive (%, if 0 positive)	27 (IQR: 12-49)	

a only cases with 12 or more lymph nodes sampled are included (N=156)

All four variants conformed to the Hardy-Weinberg equilibrium. In the entire sample (cases and controls) allele frequency of T allele was 49.55%. In the logistic regression model we found statistically significant association between rs4939827 and CRC risk. The proportion of each of the rs4939827 genotypes CC, CT and TT was 29%, 49% and 22% in controls, respectively, and 21%, 47% and 32% in cases, respectively. C allele was associated with a decreased risk, odds ratio (OR) was 0.70 (95% CI: 0.57-0.85, P=3.5E-4). Compared to TT homozygotes, risk was significantly reduced by 34% in heterozygotes (OR=0.66, 95% CI: 0.47-0.92) and by 52% in CC homozygotes (OR=0.48, 95% CI: 0.33-0.72) (Table **2**).

**Table 2:** The association between variants rs10795668, rs16892766, rs3802842 and rs4939827 and colorectal cancer in Croatian population.

**SNP**	**genotypes**	**cases**	**controls**	**effect allele**	**OR**	**95% CI:**	**P**
**rs10795668**							
	AA/AG/GG	34/123/148	48/276/270	A	0.99	0.80-1.23	0.93
	unknown	15	0				
**rs16892766**							
	AA/AC/CC	248/42/1	502/86/6	A	1.06	0.73-1.54	0.74
	unknown	29	0				
**rs3802842**							
	AA/AC/CC	132/122/17	280/252/62	A	1.15	0.92-1.45	0.23
	unknown	49	0				
**rs4939827**							
	CC/CT/TT	63/143/96	172/291/131	C	0.7	0.57-0.85	3.5x10-04
	unknown	18	0				

We next investigated the association between genotypes at rs4939827, rs10795668, rs3802842 and rs16892766 and tumour characteristics in an age and sex adjusted multivariate linear regression model. No statistically significant association was observed between selected variants and the following tumour characteristics: AJCC stage, tumour grade, tumour size or proportion of positive lymph nodes.

## Discussion

In this study we found a significant association between SMAD7 variant rs4939827 (18q21.1) and risk of colorectal cancer in Croatian population. Compared to CC homozygotes, risk was increased by 58% in heterozygotes (CT) and by 111% in TT homozygotes.

The mechanism by which rs4939827 may influence colorectal cancer involves inhibitory role of the SMAD7 protein in the transforming growth factor beta (TGF-β) signalling pathway [19,20]. TGF-β pathway regulates growth inhibition and apoptosis and plays an important role in cancer initiation and progressions [21,22].

In a comprehensive meta-analysis, we have previously shown a very high credibility of the association between rs4939827 and colorectal cancer [23]. However, in the previous studies reported effect sizes were in most part weaker than what we observed in Croatian population (OR_per T_=1.43). In a combined meta-analysis [23], found an OR of 1.15 (95% CI: 1.08-1.23) and [24] found an OR of 1.18 (95% CI: 1.14-1.22). As is reflected in overlapping confidence intervals, effect size that we observe is not significantly different than what has been previously reported. However, evidence of heterogeneity between studies exists, and further larger studies are needed to determine whether different risk estimates arise due to random variation, or they reflect true population differences.

Studies where higher OR was reported include [9] who found an OR of 1.37 (95%CI: 1.18-1.58) and [24] who found an OR of 1.57 (95% CI: 1.27-1.94) in 641 cases and 1037 controls from China [9,10,24] report a high OR in an Israeli population (OR=1.48; 95% CI: 1.33-1.65), but this may be, in part, due to poor genotyping quality (genotyping failed in >20% of samples and significant deviation from HWE was reported) [10]. The replication of GWAS signals in different ethnic groups is important as the frequency of the susceptible alleles at these loci may vary between world populations [25]. The allele frequencies we observed at rs4939827 locus in Croatian population (T allele frequency, AF_T_=49.6%) were very similar to that of the other European populations (CEU-AF_T_=44.7%; EUR-AF_T_ =52.9%), while prevalence of T allele is less frequent in Asian and African populations (ASN-AF_T_=25.9%; AFR-AF_T_ =28.7%) (http://www.ensembl.org).

Moreover, replication studies can help identify population-specific environmental factors that modify the effect of risk loci. The relatively strong association between SMAD7 variant and CRC reported here, if verified, may be due to the particular genetic and/or environmental background specific for Croatian population. This finding warrants larger studies to explore the association further, as understanding the origin of the stronger effect in this population may help uncover mechanisms of colorectal cancer occurrence and progression, and may subsequently lead to translation of findings into more accurate risk prediction and better informed prevention should the interaction with environment be found.

We failed to replicate associations between CRC risk and rs10795668, rs16892766 and rs3802842; however, this is may be due to the relatively small sample size [26-29] Similarly, small sample size may be the reason why we fail to detect significant associations between rs4939827, rs10795668, rs3802842 and rs16892766 and tumour characteristics (AJCC stage, tumour grade, tumour size or proportion of positive lymph nodes). With the exception of rs1321311, rs3824999 and rs5934683 [15], the role of other known CRC risk loci on susceptibility to CRC in Croatian population is otherwise unexplored [9-14].

Limitations of the study include relatively small sample size that may have contributed to our limited power to detect weak associations. Secondly, while controls were mainly from Split and islands of Vis and Korčula, cases were sampled from tertiary hospitals in Zagreb. However, this is not a major issue for this study: firstly, Croatian population is small and homogeneous; secondly, Zagreb and Split represent urban centres that attract immigration from other regions in Croatia, and lastly, patients would seek treatment in Zagreb due to perceived superiority of health care irrespective of their residence.

In conclusion, a common variant rs4939827 is associated with colorectal cancer risk in Croatian population. Understanding differential effects in different populations may aid in uncovering disease mechanism and is important for the translation of results to risk prediction in different populations. 

## References

[1] Tenesa A, Dunlop MG (2009) New insights into the aetiology of colorectal cancer from genome-wide association studies. Nat Rev Genet 10: 353-358. doi:10.1038/nrm2680. PubMed: 19434079. 19434079 10.1038/nrg2574

[2] Jemal A, Bray F, Center MM, Ferlay J, Ward E et al. (2011) Global cancer statistics. CA Cancer J Clin 61: 69-90. doi:10.3322/caac.20107. PubMed: 21296855. 21296855 10.3322/caac.20107

[3] Bosetti C, Levi F, Rosato V, Bertuccio P, Lucchini F et al. (2011) Recent trends in colorectal cancer mortality in Europe. Int J Cancer 129: 180-191. doi:10.1002/ijc.25653. PubMed: 20824701. 20824701 10.1002/ijc.25653

[4] Kirac I, Sekerija M, Simunović I, Zgaga L, Velimir Vrdoljak D et al. (2012) Incidence and mortality trends of gastric and colorectal cancers in Croatia, 1988-2008. Croat Med J 53: 124-134. doi:10.3325/cmj.2012.53.124. PubMed: 22522990. 22522990 10.3325/cmj.2012.53.124 PMC3342651

[5] Ferlay J, Parkin DM, Steliarova-Foucher E (2010) Estimates of cancer incidence and mortality in Europe in 2008. Eur J Cancer 46: 765-781. doi:10.1016/j.ejca.2009.12.014. PubMed: 20116997. 20116997 10.1016/j.ejca.2009.12.014

[6] TC, AM, SM, A IU, B P, et al. (2012) Deceased persons in Croatia in 2011. Zagreb: Croatian National Institute of Public Health.

[7] Katicic M, Antoljak N, Kujundzic M, Stamenic V, Skoko Poljak D et al. (2012) Results of National Colorectal Cancer Screening Program in Croatia (2007-2011). World J Gastroenterol 18: 4300-4307 10.3748/wjg.v18.i32.4300 PMC3436044 22969192

[8] Lichtenstein P, Holm NV, Verkasalo PK, Iliadou A, Kaprio J et al. (2000) Environmental and heritable factors in the causation of cancer--analyses of cohorts of twins from Sweden, Denmark, and Finland. N Engl J Med 343: 78-85. doi:10.1056/NEJM200007133430201. PubMed: 10891514. 10891514 10.1056/NEJM200007133430201

[9] Tomlinson I, Webb E, Carvajal-Carmona L, Broderick P, Kemp Z et al. (2007) A genome-wide association scan of tag SNPs identifies a susceptibility variant for colorectal cancer at 8q24.21. Nat Genet 39: 984-988. doi:10.1038/ng2085. PubMed: 17618284. 17618284 10.1038/ng2085

[10] Zanke BW, Greenwood CM, Rangrej J, Kustra R, Tenesa A et al. (2007) Genome-wide association scan identifies a colorectal cancer susceptibility locus on chromosome 8q24. Nat Genet 39: 989-994. doi:10.1038/ng2089. PubMed: 17618283. 17618283 10.1038/ng2089

[11] Houlston RS, Webb E, Broderick P, Pittman AM, Di Bernardo MC et al. (2008) Meta-analysis of genome-wide association data identifies four new susceptibility loci for colorectal cancer. Nat Genet 40: 1426-1435. doi:10.1038/ng.262. PubMed: 19011631. 19011631 10.1038/ng.262 PMC2836775

[12] Tenesa A, Farrington SM, Prendergast JG, Porteous ME, Walker M et al. (2008) Genome-wide association scan identifies a colorectal cancer susceptibility locus on 11q23 and replicates risk loci at 8q24 and 18q21. Nat Genet 40: 631-637. doi:10.1038/ng.133. PubMed: 18372901. 18372901 10.1038/ng.133 PMC2778004

[13] Tomlinson IP, Webb E, Carvajal-Carmona L, Broderick P, Howarth K et al. (2008) A genome-wide association study identifies colorectal cancer susceptibility loci on chromosomes 10p14 and 8q23.3. Nat Genet 40: 623-630. doi:10.1038/ng.111. PubMed: 18372905. 18372905 10.1038/ng.111

[14] Houlston RS, Cheadle J, Dobbins SE, Tenesa A, Jones AM et al. (2010) Meta-analysis of three genome-wide association studies identifies susceptibility loci for colorectal cancer at 1q41, 3q26.2, 12q13.13 and 20q13.33. Nat Genet 42: 973-977. doi:10.1038/ng.670. PubMed: 20972440. 20972440 10.1038/ng.670 PMC5098601

[15] Dunlop MG, Dobbins SE, Farrington SM, Jones AM, Palles C et al. (2012) Common variation near CDKN1A, POLD3 and SHROOM2 influences colorectal cancer risk. Nat Genet 44: 770-776. doi:10.1038/ng.2293. PubMed: 22634755. 22634755 10.1038/ng.2293 PMC4747430

[16] Ioannidis JP (2007) Non-replication and inconsistency in the genome-wide association setting. Hum Hered 64: 203-213. doi:10.1159/000103512. PubMed: 17551261. 17551261 10.1159/000103512

[17] Sawyer SL, Mukherjee N, Pakstis AJ, Feuk L, Kidd JR et al. (2005) Linkage disequilibrium patterns vary substantially among populations. Eur J Hum Genet 13: 677-686. doi:10.1038/sj.ejhg.5201368. PubMed: 15657612. 15657612 10.1038/sj.ejhg.5201368

[18] Edge SB, Byrd DR, Compton CC, Fritz AG, Greene FL et al. (2009) AJCC Cancer Staging Manual. New York: Springer Verlag. 646pp.

[19] Loh YH, Mitrou PN, Wood A, Luben RN, McTaggart A et al. (2011) SMAD7 and MGMT genotype variants and cancer incidence in the European Prospective Investigation into Cancer and Nutrition (EPIC)-Norfolk Study. Cancer Epidemiol 35: 369-374. doi:10.1016/j.canep.2010.09.011. PubMed: 21075068. 21075068 10.1016/j.canep.2010.09.011

[20] Zhong R, Liu L, Zou L, Sheng W, Zhu B et al. (2013) Genetic variations in the TGFbeta signaling pathway, smoking and risk of colorectal cancer in a Chinese population. Carcinogenesis 34: 936-942. doi:10.1093/carcin/bgs395. PubMed: 23275154. 23275154 10.1093/carcin/bgs395

[21] Halder SK, Beauchamp RD, Datta PK (2005) Smad7 induces tumorigenicity by blocking TGF-beta-induced growth inhibition and apoptosis. Exp Cell Res 307: 231-246. doi:10.1016/j.yexcr.2005.03.009. PubMed: 15922743. 15922743 10.1016/j.yexcr.2005.03.009

[22] Siegel PM, Massagué J (2003) Cytostatic and apoptotic actions of TGF-beta in homeostasis and cancer. Nat Rev Cancer 3: 807-821. doi:10.1038/nrc1208. PubMed: 14557817. 14557817 10.1038/nrc1208

[23] Theodoratou E, Montazeri Z, Hawken S, Allum GC, Gong J et al. (2012) Systematic meta-analyses and field synopsis of genetic association studies in colorectal cancer. J Natl Cancer Inst 104: 1433-1457. doi:10.1093/jnci/djs369. PubMed: 23019048. 23019048 10.1093/jnci/djs369

[24] Song Q, Zhu B, Hu W, Cheng L, Gong H et al. (2012) A common SMAD7 variant is associated with risk of colorectal cancer: evidence from a case-control study and a meta-analysis. PLOS ONE 7: e33318. doi:10.1371/journal.pone.0033318. PubMed: 22457752. 22457752 10.1371/journal.pone.0033318 PMC3310071

[25] Kruglyak L (1999) Genetic isolates: separate but equal? Proc Natl Acad Sci U S A 96: 1170-1172. doi:10.1073/pnas.96.4.1170. PubMed: 9989995. 9989995 10.1073/pnas.96.4.1170 PMC33550

[26] Poynter JN, Figueiredo JC, Conti DV, Kennedy K, Gallinger S et al. (2007) Variants on 9p24 and 8q24 are associated with risk of colorectal cancer: results from the Colon Cancer Family Registry. Cancer Res 67: 11128-11132. doi:10.1158/0008-5472.CAN-07-3239. PubMed: 18056436. 18056436 10.1158/0008-5472.CAN-07-3239

[27] Middeldorp A, Jagmohan-Changur S, van Eijk R, Tops C, Devilee P et al. (2009) Enrichment of low penetrance susceptibility loci in a Dutch familial colorectal cancer cohort. Cancer epidemiology, biomarkers & prevention : a publication of the American Association for Cancer Research, cosponsored by the American Society of Preventive Oncology 18: 3062-3067 10.1158/1055-9965.EPI-09-0601 19843678

[28] von Holst S, Picelli S, Edler D, Lenander C, Dalén J et al. (2010) Association studies on 11 published colorectal cancer risk loci. Br J Cancer 103: 575-580. doi:10.1038/sj.bjc.6605774. PubMed: 20648012. 20648012 10.1038/sj.bjc.6605774 PMC2939779

[29] Thompson CL, Plummer SJ, Acheson LS, Tucker TC, Casey G et al. (2009) Association of common genetic variants in SMAD7 and risk of colon cancer. Carcinogenesis 30: 982-986. doi:10.1093/carcin/bgp086. PubMed: 19357349. 19357349 10.1093/carcin/bgp086 PMC2691142

